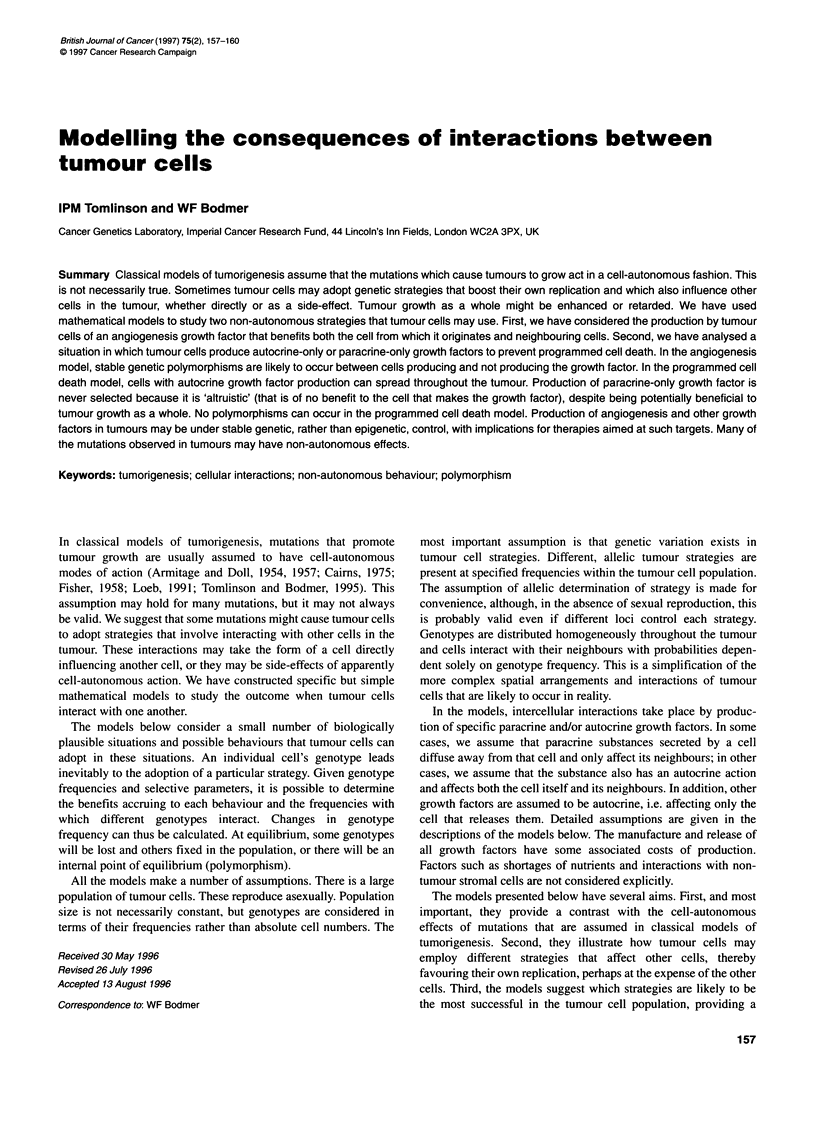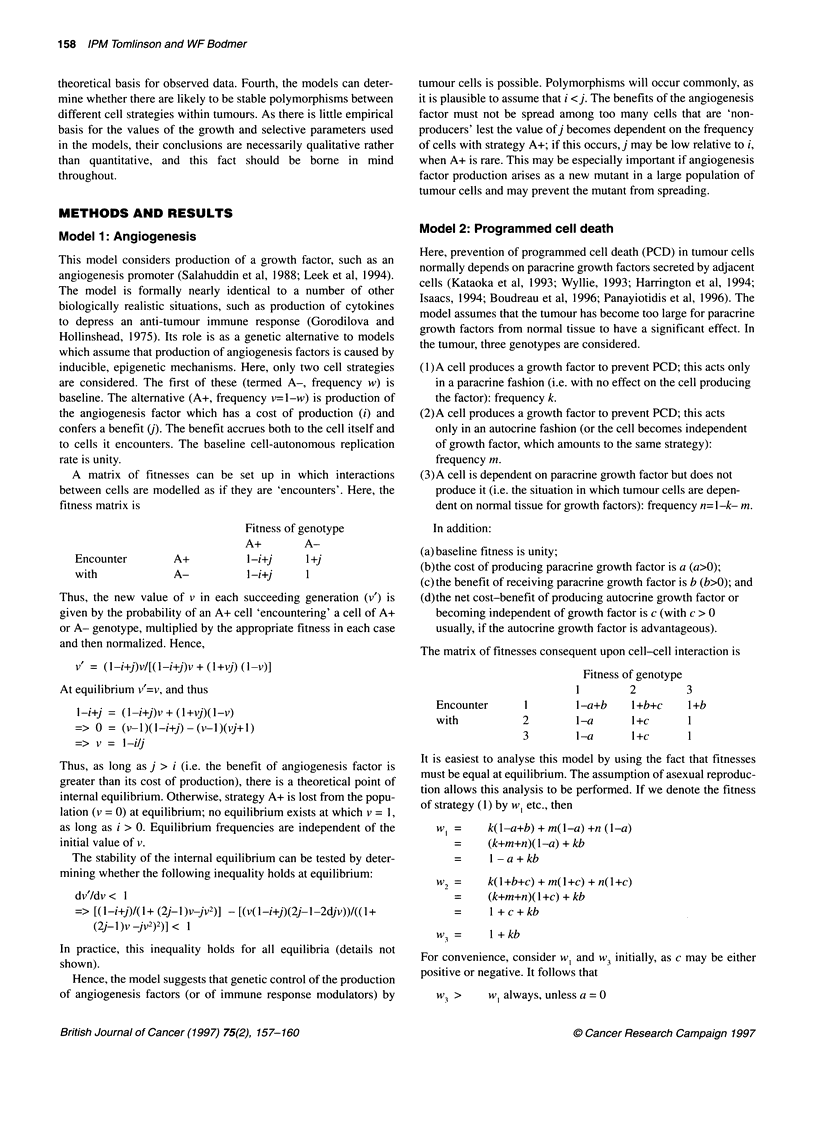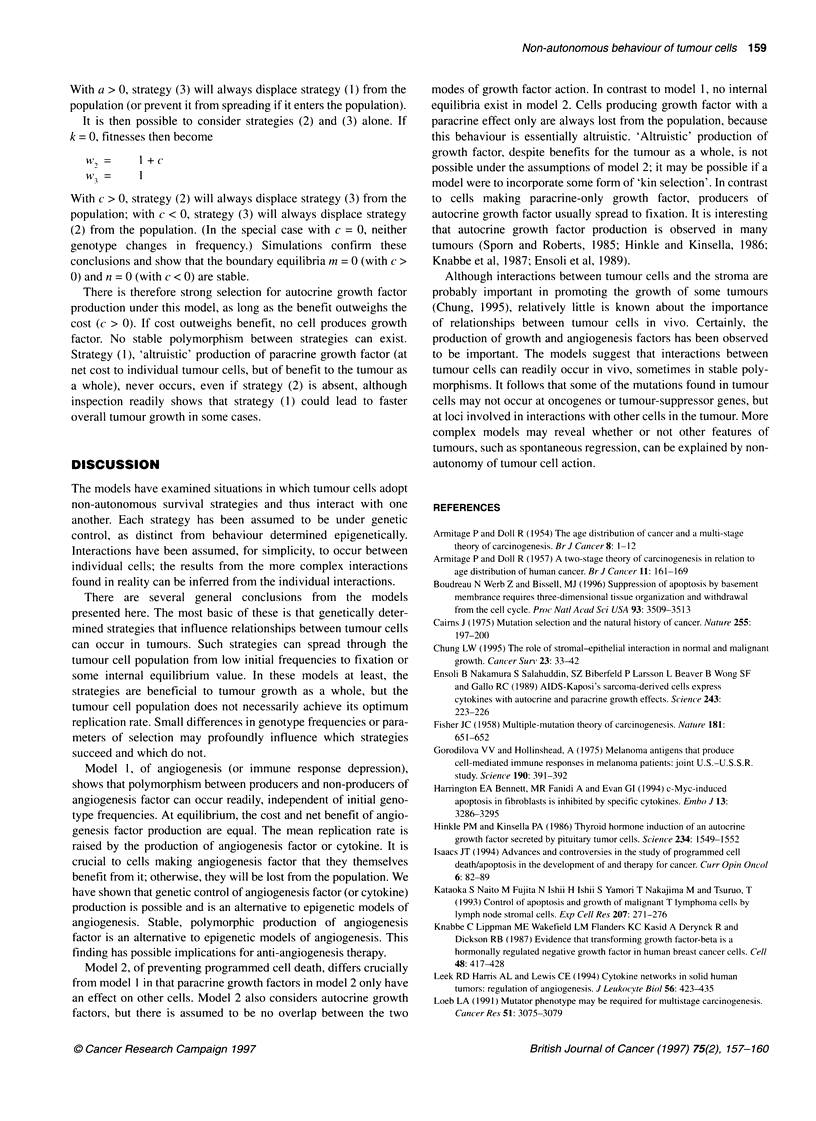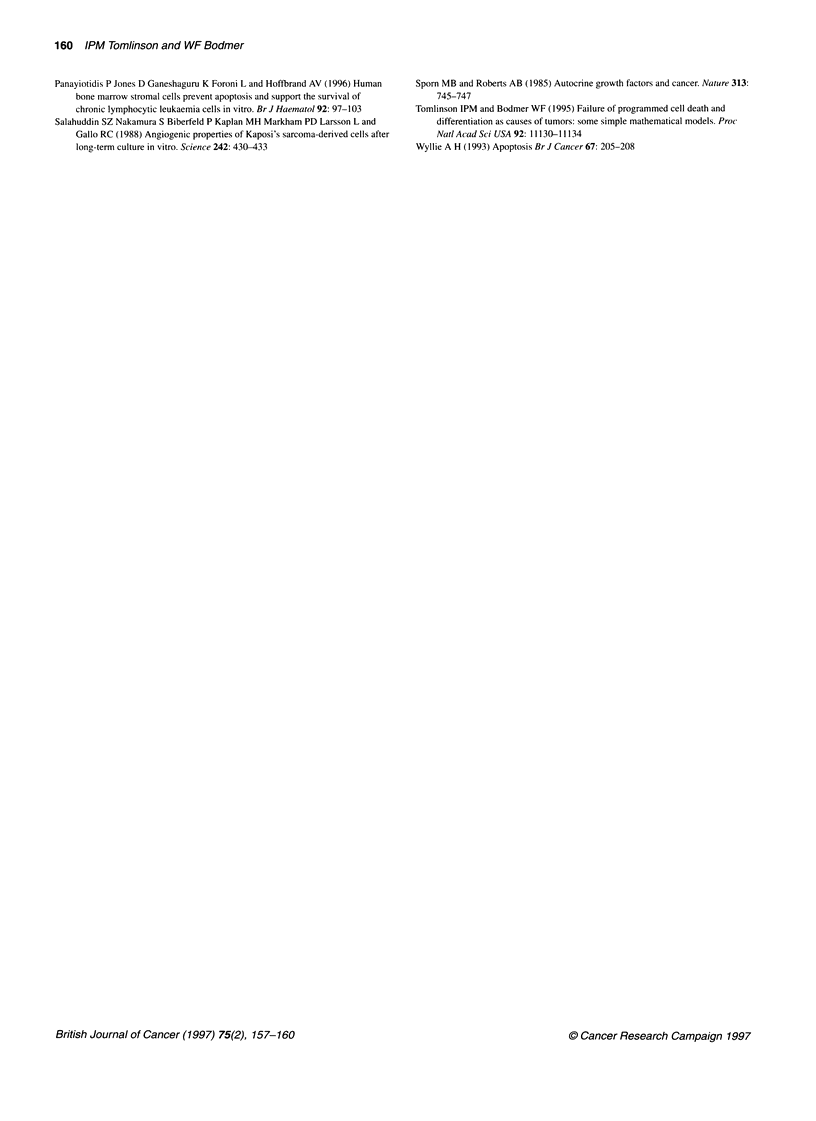# Modelling the consequences of interactions between tumour cells.

**DOI:** 10.1038/bjc.1997.26

**Published:** 1997

**Authors:** I. P. Tomlinson, W. F. Bodmer

**Affiliations:** Cancer Genetics Laboratory, Imperial Cancer Research Fund, London, UK.

## Abstract

Classical models of tumorigenesis assume that the mutations which cause tumours to grow act in a cell-autonomous fashion. This is not necessarily true. Sometimes tumour cells may adopt genetic strategies that boost their own replication and which also influence other cells in the tumour, whether directly or as a side-effect. Tumour growth as a whole might be enhanced or retarded. We have used mathematical models to study two non-autonomous strategies that tumour cells may use. First, we have considered the production by tumour cells of an angiogenesis growth factor that benefits both the cell from which it originates and neighbouring cells. Second, we have analysed a situation in which tumour cells produce autocrine-only or paracrine-only growth factors to prevent programmed cell death. In the angiogenesis model, stable genetic polymorphisms are likely to occur between cells producing and not producing the growth factor. In the programmed cell death model, cells with autocrine growth factor production can spread throughout the tumour. Production of paracrine-only growth factor is never selected because it is 'altruistic' (that is of no benefit to the cell that makes the growth factor), despite being potentially beneficial to tumour growth as a whole. No polymorphisms can occur in the programmed cell death model. Production of angiogenesis and other growth factors in tumours may be under stable genetic, rather than epigenetic, control, with implications for therapies aimed at such targets. Many of the mutations observed in tumours may have non-autonomous effects.


*British Journal of Cancer* (1997) **75**(2), 157–160
© 1997 Cancer Research Campaign

### Modelling the consequences of interactions between tumour cells

**IPM Tomlinson and WF Bodmer**

Cancer Genetics Laboratory, Imperial Cancer Research Fund, 44 Lincoln's Inn Fields, London WC2A 3PX, UK

**Summary** Classical models of tumorigenesis assume that the mutations which cause tumours to grow act in a cell-autonomous fashion. This is not necessarily true. Sometimes tumour cells may adopt genetic strategies that boost their own replication and which also influence other cells in the tumour, whether directly or as a side-effect. Tumour growth as a whole might be enhanced or retarded. We have used mathematical models to study two non-autonomous strategies that tumour cells may use. First, we have considered the production by tumour cells of an angiogenesis growth factor that benefits both the cell from which it originates and neighbouring cells. Second, we have analysed a situation in which tumour cells produce autocrine-only or paracrine-only growth factors to prevent programmed cell death. In the angiogenesis model, stable genetic polymorphisms are likely to occur between cells producing and not producing the growth factor. In the programmed cell death model, cells with autocrine growth factor production can spread throughout the tumour. Production of paracrine-only growth factor is never selected because it is 'altruistic' (that is of no benefit to the cell that makes the growth factor), despite being potentially beneficial to tumour growth as a whole. No polymorphisms can occur in the programmed cell death model. Production of angiogenesis and other growth factors in tumours may be under stable genetic, rather than epigenetic, control, with implications for therapies aimed at such targets. Many of the mutations observed in tumours may have non-autonomous effects.

**Keywords:** tumorigenesis; cellular interactions; non-autonomous behaviour; polymorphism

In classical models of tumorigenesis, mutations that promote tumour growth are usually assumed to have cell-autonomous modes of action (Armitage and Doll, 1954, 1957; Cairns, 1975; Fisher, 1958; Loeb, 1991; Tomlinson and Bodmer, 1995). This assumption may hold for many mutations, but it may not always be valid. We suggest that some mutations might cause tumour cells to adopt strategies that involve interacting with other cells in the tumour. These interactions may take the form of a cell directly influencing another cell, or they may be side-effects of apparently cell-autonomous action. We have constructed specific but simple mathematical models to study the outcome when tumour cells interact with one another.

The models below consider a small number of biologically plausible situations and possible behaviours that tumour cells can adopt in these situations. An individual cell's genotype leads inevitably to the adoption of a particular strategy. Given genotype frequencies and selective parameters, it is possible to determine the benefits accruing to each behaviour and the frequencies with which different genotypes interact. Changes in genotype frequency can thus be calculated. At equilibrium, some genotypes will be lost and others fixed in the population, or there will be an internal point of equilibrium (polymorphism).

All the models make a number of assumptions. There is a large population of tumour cells. These reproduce asexually. Population size is not necessarily constant, but genotypes are considered in terms of their frequencies rather than absolute cell numbers. The most important assumption is that genetic variation exists in tumour cell strategies. Different, allelic tumour strategies are present at specified frequencies within the tumour cell population. The assumption of allelic determination of strategy is made for convenience, although, in the absence of sexual reproduction, this is probably valid even if different loci control each strategy. Genotypes are distributed homogeneously throughout the tumour and cells interact with their neighbours with probabilities dependent solely on genotype frequency. This is a simplification of the more complex spatial arrangements and interactions of tumour cells that are likely to occur in reality.

In the models, intercellular interactions take place by production of specific paracrine and/or autocrine growth factors. In some cases, we assume that paracrine substances secreted by a cell diffuse away from that cell and only affect its neighbours; in other cases, we assume that the substance also has an autocrine action and affects both the cell itself and its neighbours. In addition, other growth factors are assumed to be autocrine, i.e. affecting only the cell that releases them. Detailed assumptions are given in the descriptions of the models below. The manufacture and release of all growth factors have some associated costs of production. Factors such as shortages of nutrients and interactions with non-tumour stromal cells are not considered explicitly.

The models presented below have several aims. First, and most important, they provide a contrast with the cell-autonomous effects of mutations that are assumed in classical models of tumorigenesis. Second, they illustrate how tumour cells may employ different strategies that affect other cells, thereby favouring their own replication, perhaps at the expense of the other cells. Third, the models suggest which strategies are likely to be the most successful in the tumour cell population, providing a

*Received 30 May 1996*
*Revised 26 July 1996*
*Accepted 13 August 1996*

*Correspondence to*: WF Bodmer theoretical basis for observed data. Fourth, the models can determine whether there are likely to be stable polymorphisms between different cell strategies within tumours. As there is little empirical basis for the values of the growth and selective parameters used in the models, their conclusions are necessarily qualitative rather than quantitative, and this fact should be borne in mind throughout.

### METHODS AND RESULTS

### Model 1: Angiogenesis

This model considers production of a growth factor, such as an angiogenesis promoter (Salahuddin et al, 1988; Leek et al, 1994). The model is formally nearly identical to a number of other biologically realistic situations, such as production of cytokines to depress an anti-tumour immune response (Gorodilova and Hollinshead, 1975). Its role is as a genetic alternative to models which assume that production of angiogenesis factors is caused by inducible, epigenetic mechanisms. Here, only two cell strategies are considered. The first of these (termed A–, frequency $w$) is baseline. The alternative (A+, frequency $v=1-w$) is production of the angiogenesis factor which has a cost of production ($i$) and confers a benefit ($j$). The benefit accrues both to the cell itself and to cells it encounters. The baseline cell-autonomous replication rate is unity.

A matrix of fitnesses can be set up in which interactions between cells are modelled as if they are 'encounters'. Here, the fitness matrix is

| | | Fitness of genotype | |
|---|---|---|---|
| | | A+ | A– |
| Encounter | A+ | $1-i+j$ | $1+j$ |
| with | A– | $1-i+j$ | 1 |

Thus, the new value of $v$ in each succeeding generation ($v'$) is given by the probability of an A+ cell 'encountering' a cell of A+ or A– genotype, multiplied by the appropriate fitness in each case and then normalized. Hence,

$$v' = (1-i+j)v/[(1-i+j)v + (1+vj)(1-v)]$$

At equilibrium $v'=v$, and thus

$$1-i+j = (1-i+j)v + (1+vj)(1-v)$$
$$\Rightarrow 0 = (v-1)(1-i+j) - (v-1)(vj+1)$$
$$\Rightarrow v = 1-i/j$$

Thus, as long as $j > i$ (i.e. the benefit of angiogenesis factor is greater than its cost of production), there is a theoretical point of internal equilibrium. Otherwise, strategy A+ is lost from the population ($v = 0$) at equilibrium; no equilibrium exists at which $v = 1$, as long as $i > 0$. Equilibrium frequencies are independent of the initial value of $v$.

The stability of the internal equilibrium can be tested by determining whether the following inequality holds at equilibrium:

$$dv'/dv < 1$$

$$\Rightarrow [(1-i+j)/(1+(2j-1)v-jv^2)] - [(v(1-i+j)(2j-1-2djv))/((1+(2j-1)v-jv^2)^2)] < 1$$

In practice, this inequality holds for all equilibria (details not shown).

Hence, the model suggests that genetic control of the production of angiogenesis factors (or of immune response modulators) by tumour cells is possible. Polymorphisms will occur commonly, as it is plausible to assume that $i < j$. The benefits of the angiogenesis factor must not be spread among too many cells that are 'non-producers' lest the value of $j$ becomes dependent on the frequency of cells with strategy A+; if this occurs, $j$ may be low relative to $i$, when A+ is rare. This may be especially important if angiogenesis factor production arises as a new mutant in a large population of tumour cells and may prevent the mutant from spreading.

### Model 2: Programmed cell death

Here, prevention of programmed cell death (PCD) in tumour cells normally depends on paracrine growth factors secreted by adjacent cells (Kataoka et al, 1993; Wyllie, 1993; Harrington et al, 1994; Isaacs, 1994; Boudreau et al, 1996; Panayiotidis et al, 1996). The model assumes that the tumour has become too large for paracrine growth factors from normal tissue to have a significant effect. In the tumour, three genotypes are considered.

(1) A cell produces a growth factor to prevent PCD; this acts only in a paracrine fashion (i.e. with no effect on the cell producing the factor): frequency $k$.
(2) A cell produces a growth factor to prevent PCD; this acts only in an autocrine fashion (or the cell becomes independent of growth factor, which amounts to the same strategy): frequency $m$.
(3) A cell is dependent on paracrine growth factor but does not produce it (i.e. the situation in which tumour cells are dependent on normal tissue for growth factors): frequency $n=1-k-m$.

In addition:

(a) baseline fitness is unity;
(b) the cost of producing paracrine growth factor is $a$ ($a$>0);
(c) the benefit of receiving paracrine growth factor is $b$ ($b$>0); and
(d) the net cost–benefit of producing autocrine growth factor or becoming independent of growth factor is $c$ (with $c > 0$ usually, if the autocrine growth factor is advantageous).

The matrix of fitnesses consequent upon cell–cell interaction is

| | | Fitness of genotype | | |
|---|---|---|---|---|
| | | 1 | 2 | 3 |
| Encounter | 1 | $1-a+b$ | $1+b+c$ | $1+b$ |
| with | 2 | $1-a$ | $1+c$ | 1 |
| | 3 | $1-a$ | $1+c$ | 1 |

It is easiest to analyse this model by using the fact that fitnesses must be equal at equilibrium. The assumption of asexual reproduction allows this analysis to be performed. If we denote the fitness of strategy (1) by $w_1$ etc., then

$$w_1 = k(1-a+b) + m(1-a) + n(1-a) = (k+m+n)(1-a) + kb = 1 - a + kb$$

$$w_2 = k(1+b+c) + m(1+c) + n(1+c) = (k+m+n)(1+c) + kb = 1 + c + kb$$

$$w_3 = 1 + kb$$

For convenience, consider $w_1$ and $w_3$ initially, as $c$ may be either positive or negative. It follows that

$$w_3 > w_1 \text{ always, unless } a = 0$$

© Cancer Research Campaign 1997

With $a > 0$, strategy (3) will always displace strategy (1) from the population (or prevent it from spreading if it enters the population).

It is then possible to consider strategies (2) and (3) alone. If $k = 0$, fitnesses then become

$$w_2 = 1 + c$$
$$w_3 = 1$$

With $c > 0$, strategy (2) will always displace strategy (3) from the population; with $c < 0$, strategy (3) will always displace strategy (2) from the population. (In the special case with $c = 0$, neither genotype changes in frequency.) Simulations confirm these conclusions and show that the boundary equilibria $m = 0$ (with $c >$ 0) and $n = 0$ (with $c < 0$) are stable.

There is therefore strong selection for autocrine growth factor production under this model, as long as the benefit outweighs the cost ($c > 0$). If cost outweighs benefit, no cell produces growth factor. No stable polymorphism between strategies can exist. Strategy (1), 'altruistic' production of paracrine growth factor (at net cost to individual tumour cells, but of benefit to the tumour as a whole), never occurs, even if strategy (2) is absent, although inspection readily shows that strategy (1) could lead to faster overall tumour growth in some cases.

### DISCUSSION

The models have examined situations in which tumour cells adopt non-autonomous survival strategies and thus interact with one another. Each strategy has been assumed to be under genetic control, as distinct from behaviour determined epigenetically. Interactions have been assumed, for simplicity, to occur between individual cells; the results from the more complex interactions found in reality can be inferred from the individual interactions.

There are several general conclusions from the models presented here. The most basic of these is that genetically determined strategies that influence relationships between tumour cells can occur in tumours. Such strategies can spread through the tumour cell population from low initial frequencies to fixation or some internal equilibrium value. In these models at least, the strategies are beneficial to tumour growth as a whole, but the tumour cell population does not necessarily achieve its optimum replication rate. Small differences in genotype frequencies or parameters of selection may profoundly influence which strategies succeed and which do not.

Model 1, of angiogenesis (or immune response depression), shows that polymorphism between producers and non-producers of angiogenesis factor can occur readily, independent of initial genotype frequencies. At equilibrium, the cost and net benefit of angiogenesis factor production are equal. The mean replication rate is raised by the production of angiogenesis factor or cytokine. It is crucial to cells making angiogenesis factor that they themselves benefit from it; otherwise, they will be lost from the population. We have shown that genetic control of angiogenesis factor (or cytokine) production is possible and is an alternative to epigenetic models of angiogenesis. Stable, polymorphic production of angiogenesis factor is an alternative to epigenetic models of angiogenesis. This finding has possible implications for anti-angiogenesis therapy.

Model 2, of preventing programmed cell death, differs crucially from model 1 in that paracrine growth factors in model 2 only have an effect on other cells. Model 2 also considers autocrine growth factors, but there is assumed to be no overlap between the two modes of growth factor action. In contrast to model 1, no internal equilibria exist in model 2. Cells producing growth factor with a paracrine effect only are always lost from the population, because this behaviour is essentially altruistic. 'Altruistic' production of growth factor, despite benefits for the tumour as a whole, is not possible under the assumptions of model 2; it may be possible if a model were to incorporate some form of 'kin selection'. In contrast to cells making paracrine-only growth factor, producers of autocrine growth factor usually spread to fixation. It is interesting that autocrine growth factor production is observed in many tumours (Sporn and Roberts, 1985; Hinkle and Kinsella, 1986; Knabbe et al, 1987; Ensoli et al, 1989).

Although interactions between tumour cells and the stroma are probably important in promoting the growth of some tumours (Chung, 1995), relatively little is known about the importance of relationships between tumour cells in vivo. Certainly, the production of growth and angiogenesis factors has been observed to be important. The models suggest that interactions between tumour cells can readily occur in vivo, sometimes in stable polymorphisms. It follows that some of the mutations found in tumour cells may not occur at oncogenes or tumour-suppressor genes, but at loci involved in interactions with other cells in the tumour. More complex models may reveal whether or not other features of tumours, such as spontaneous regression, can be explained by non-autonomy of tumour cell action.

### REFERENCES

Armitage P and Doll R (1954) The age distribution of cancer and a multi-stage theory of carcinogenesis. *Br J Cancer* **8**: 1–12

Armitage P and Doll R (1957) A two-stage theory of carcinogenesis in relation to age distribution of human cancer. *Br J Cancer* **11**: 161–169

Boudreau N Werb Z and Bissell, MJ (1996) Suppression of apoptosis by basement membrance requires three-dimensional tissue organization and withdrawal from the cell cycle. *Proc Natl Acad Sci USA* **93**: 3509–3513

Cairns J (1975) Mutation selection and the natural history of cancer. *Nature* **255**: 197–200

Chung LW (1995) The role of stromal–epithelial interaction in normal and malignant growth. *Cancer Surv* **23**: 33–42

Ensoli B Nakamura S Salahuddin, SZ Biberfeld P Larsson L Beaver B Wong SF and Gallo RC (1989) AIDS-Kaposi's sarcoma-derived cells express cytokines with autocrine and paracrine growth effects. *Science* **243**: 223–226

Fisher JC (1958) Multiple-mutation theory of carcinogenesis. *Nature* **181**: 651–652

Gorodilova VV and Hollinshead, A (1975) Melanoma antigens that produce cell-mediated immune responses in melanoma patients: joint U.S.–U.S.S.R. study. *Science* **190**: 391–392

Harrington EA Bennett, MR Fanidi A and Evan GI (1994) c-Myc-induced apoptosis in fibroblasts is inhibited by specific cytokines. *Embo J* **13**: 3286–3295

Hinkle PM and Kinsella PA (1986) Thyroid hormone induction of an autocrine growth factor secreted by pituitary tumor cells. *Science* **234**: 1549–1552

Isaacs JT (1994) Advances and controversies in the study of programmed cell death/apoptosis in the development of and therapy for cancer. *Curr Opin Oncol* **6**: 82–89

Kataoka S Naito M Fujita N Ishii H Ishii S Yamori T Nakajima M and Tsuruo, T (1993) Control of apoptosis and growth of malignant T lymphoma cells by lymph node stromal cells. *Exp Cell Res* **207**: 271–276

Knabbe C Lippman ME Wakefield LM Flanders KC Kasid A Derynck R and Dickson RB (1987) Evidence that transforming growth factor-beta is a hormonally regulated negative growth factor in human breast cancer cells. *Cell* **48**: 417–428

Leek RD Harris AL and Lewis CE (1994) Cytokine networks in solid human tumors: regulation of angiogenesis. *J Leukocyte Biol* **56**: 423–435

Loeb LA (1991) Mutator phenotype may be required for multistage carcinogenesis. *Cancer Res* **51**: 3075–3079

© Cancer Research Campaign 1997

Panayiotidis P Jones D Ganeshaguru K Foroni L and Hoffbrand AV (1996) Human bone marrow stromal cells prevent apoptosis and support the survival of chronic lymphocytic leukaemia cells in vitro. *Br J Haematol* **92**: 97–103

Salahuddin SZ Nakamura S Biberfeld P Kaplan MH Markham PD Larsson L and Gallo RC (1988) Angiogenic properties of Kaposi's sarcoma-derived cells after long-term culture in vitro. *Science* **242**: 430–433

Sporn MB and Roberts AB (1985) Autocrine growth factors and cancer. *Nature* **313**: 745–747

Tomlinson IPM and Bodmer WF (1995) Failure of programmed cell death and differentiation as causes of tumors: some simple mathematical models. *Proc Natl Acad Sci USA* **92**: 11130–11134

Wyllie A H (1993) Apoptosis *Br J Cancer* **67**: 205–208

 *© Cancer Research Campaign 1997*